# Supplementary material for: Social-Ecological Correlates of Regular Leisure-Time Physical Activity Practice among Adults
Source: Int J Environ Res Public Health. 2020 May 21;17(10):3619. doi: 10.3390/ijerph17103619 (PMC7277494; doi:10.3390/ijerph17103619)
Supplement: Supplementary file 1 [file ijerph-17-03619-s001.pdf]

Supplementary Material

Table SM1 – Sample data VIGITEL 2006-2016

	TELEPHONES (n)			INTERVIEWS (n)		
**Year**	**Replica**	**Randomly Selected**	**Elegible**	**Total**	**Men**	**Women**
2006	536	107,200	76,330	54,369	21,294	33,075
2007	693	138,600	75,876	54,251	21,547	32,704
2008	530	106,000	72,834	54,353	21,345	32,918
2009	591	118,200	71,081	54,367	21,347	33,020
2010	633	126,600	71,082	54,339	20,764	33,575
2011	576	111,200	80,470	54,144	20,641	31,503
2012	577	115,418	70,045	45,488	17,389	28,059
2013	563	112,600	74,005	52,929	20,272	32,653
2014	506	101,200	62,786	40,853	15,521	25,332
2015	580	116,000	76,703	54,174	20,368	32,653
2016	636	127,200	77,671	53,210	20,258	32,952
**TOTAL**	**6.421**	**1,280,218**	**808,883**	**572,477**	**220,746**	**348,444**

Source: VIGITEL 2006-2016 (IBGE – www.ibge.com.br)

Table SM2 – Data extracted, unit of measure and Source

INFORMAÇÃO	Unit	Source
Precipitação	mm	INMET
Insolação	Horas	INMET
Temperatura mínima	Graus Celsius	INMET
Temperatura máxima	Graus Celsius	INMET
Umidade relativa	%	INMET
Umidade mínima	%	INMET
Umidade mínima	%	INMET
Frota de veículos	n	DENATRAN
Frota de automóveis	n	DENATRAN
Frota de ônibus	n	DENATRAN
Nº de empresas de esporte e lazer	n	IBGE/CCE
Idade	years	VIGITEL
Sexo	m/f	VIGITEL
Marital status	s/m/d/w/na***	VIGITEL
Uso de tabaco	s/n	VIGITEL
Excesso de peso	s/n	VIGITEL
Obesidade	s/n	VIGITEL
Consumo de hortaliças regularmente	s/n	VIGITEL
Consumo de frutas regularmente	s/n	VIGITEL
Consumo de frutas legumes e verduras regularmente	s/n	VIGITEL
Consumo de carne regularmente	s/n	VIGITEL
Consumo de pele de frango	s/n	VIGITEL
Consumo de gordura da carne	s/n	VIGITEL
Consumo de leite integral	s/n	VIGITEL
Consumo de refrigerante	s/n	VIGITEL
Atividade física tempo livre	s/n	VIGITEL
Consumo abusivo de álcool	s/n	VIGITEL
Saúde autorrelatada (ruim)	s/n	VIGITEL
Hipertensão arterial sistêmica	s/n	VIGITEL
Diabetes	s/n	VIGITEL
Tempo assistindo TV acima de 3 horas diárias	s/n	VIGITEL
PIB bruto	R$	IBGE
PIB per capita	R$	IBGE
Valor da cesta básica	R$	PNCBA
Índice de GINI para desigualdade	Coeficiente (0-1)	DATASUS
Cobertura do abastecimento de água	%	IBGE
Investimentos em esporte e lazer	BR$	STN
Investimentos em saúde	BR$	STN
Cobertura de planos de saúde	n	DATASUS
Cobertura de equipes de atenção básica	n	DATASUS
Cobertura de estratégia de saúde da família	n	DATASUS
Expectativa de vida	years	IBGE
Mortalidade por crime	n	IBGE
Mortalidade por acidente	n	IBGE
Número de habitantes	n	IBGE
Número de ocupados em esporte e lazer	n	IBGE/CCE
Famílias com renda inferior a meio salário mínimo	%	IBGE
Famílias com renda entre 1/2 e 1 salário mínimo	%	IBGE
Famílias com renda entre 1 e 2 salários mínimos	%	IBGE
Famílias com renda superior a 2 salários mínimos	%	IBGE

***Marital status: s: single; m: married; d: divorced; w: widower; na: no answer.

Table SM3 – Cut-off for social-ecological correlates into Tertiles

	1º TERCIL	2º TERCIL	3º TERCIL
**CONDIÇÃO**	≥ ; <	≥ ; ≤	> ; ≤
Age	18 ; 33	33 ; 45	45 ; 59
Crime Mortality (n/100,000 inhab,)	1.05 ; 25.9	25.9 ; 99.5	99.5 ; 1100
Traffic Accident Mortality (n/100,000 inhab,)	0.618 ; 15.8	15.8 ; 44.1	44.1 ; 482
Population (milhões)	0.184 ; 0.585	0.585 ; 1.47	1.47 ; 12
Female Proportion (%)	0.474 ; 0.52	0.52 ; 0.531	0.531 ; 0.54
Vehicle Fleet (milions)	0.0554 ; 0.218	0.218 ; 0.548	0.548 ; 7.81
Car Fleet (n/100,000 inhab,)	12500 ; 28700	28700 ; 44100	44100 ; 74100
Bus Fleet (n/100,000 inhab,)	143 ; 360	360 ; 498	498 ; 792
GINI (UA)	0.419 ; 0.5	0.5 ; 0.541	0.541 ; 0.666
Private Health Insurance (n/100,000 inhab,)	5990 ; 24100	24100 ; 35300	35300 ; 77500
GDP (milhões BR$)	2.04 ; 11.9	11.9 ; 31.6	31.6 ; 687
GDP Per capita (BR$/per capita)	7680 ; 17100	17100 ; 25000	25000 ; 79100
Employees Physical Activity (inhab/n)	195 ; 484	484 ; 1030	1030 ; 2910
Company Physical Activity (inhab,/n)	648 ; 1970	1970 ; 3510	3510 ; 7800
Insolation (h/year)	475 ; 2200	2200 ; 2430	2430 ; 3250
Rainfall (mm/year)	104 ; 1560	1560 ; 1910	1910 ; 3780
Max Temperature (º C)	23 ; 29.7	29.7 ; 31.7	31.7 ; 35.5
Min Temperatures (º C)	13.4 ; 21.3	21.3 ; 23.1	23.1 ; 25.7
Relative Humidity (%)	56.2 ; 74.8	74.8 ; 78.9	78.9 ; 98
Min Relative Humidity (%)	31 ; 66.1	66.1 ; 73.1	73.1 ; 98
Max Relative Humidity (%)	62.6 ; 81.9	81.9 ; 85.3	85.3 ; 98
Life expectancy (years)	67.3 ; 71.2	71.2 ; 73.8	73.8 ; 79.1
Male Life expectancy (years)	63 ; 67.7	67.7 ; 70.4	70.4 ; 75.8
Female Life expectancy (years)	71.2 ; 75.1	75.1 ; 77.5	77.5 ; 82.4
FHS Public Health (n)	41 ; 98	98 ; 209	209 ; 1530
Primary Care Coverage (%)	20.9 ; 53	53 ; 70.2	70.2 ; 100
Public Investment* in Sports And Leisure Percapita (R$/per capita)	0.000562 ; 4.18	4.18 ; 10.5	10.5 ; 118
Public Investment* in Health (R$/per capita)	118 ; 350	350 ; 550	550 ; 1260
Drinking Water Distribuition (%)	49.2 ; 89.8	89.8 ; 95.9	95.9 ; 99.3
Basic Grocery Package (R$)	132.0 ; 213.0	213.0 ; 272.0	272.0 ; 459.0
Family Income < 1/2 min Wage/month (%)	5.2 ; 16.7	16.7 ; 31.2	31.2 ; 47.1
Family Income 1/2 to 1 min Wage//month (%)	18 ; 29	29 ; 31.2	31.2 ; 36.4
Family Income 1 to 2 min Wage//month (%)	13.2 ; 20.4	20.4 ; 27.6	27.6 ; 38.6
Family Income >2 min Wage//month (%)	7.9 ; 13.6	13.6 ; 21.2	21.2 ; 43.9

Table SM4 – Encouraging social-ecological factors detection using complex samples logistical regression

	MEN									WOMEN								
	Age 18-32			Age 33-45			Age 46-60			Age 18-32			Age 33-45			Age 46-60		
**LABELS**	**OR**	**95% CI**		**OR**	**95% CI**		**OR**	**95% CI**		**OR**	**95% CI**		**OR**	**95% CI**		**OR**	**95% CI**	
TABACO USE (n vs, y)	0.925	0.786	1.089	0.663	0.54	0.814	0.681	0.567	0.818	0.984	0.757	1.279	0.807	0.633	1.028	0.747	0.627	0.89
OBESITY (n vs, y)	1.297	1.125	1.496	1.088	0.946	1.252	1.243	1.081	1.429	1.021	0.859	1.213	1.238	1.078	1.422	1.257	1.114	1.417
VEGETABLES (y vs, n)	1.286	1.109	1.491	1.531	1.275	1.838	1.54	1.263	1.878	1.615	1.351	1.93	1.708	1.395	2.092	1.457	1.164	1.823
FRUITS (y vs, n)	1.667	1.473	1.886	1.457	1.23	1.725	1.732	1.456	2.061	1.61	1.38	1.879	1.917	1.606	2.287	1.754	1.452	2.118
FLV (y vs, n)	1.066	0.885	1.285	1.054	0.837	1.328	0.942	0.742	1.195	0.974	0.788	1.204	0.804	0.638	1.014	1.033	0.806	1.324
MEAT (n vs, y)	1.011	0.849	1.205	1.005	0.789	1.278	1.056	0.815	1.369	1.002	0.753	1.332	1.259	0.91	1.743	1.538	1.072	2.206
CHICKEN SKIN (n vs, y)	0.845	0.72	0.991	0.984	0.797	1.215	1.192	0.936	1.518	1.27	0.986	1.635	1.167	0.869	1.568	1.601	1.148	2.233
MEAT FAT (n vs, y)	1.196	0.968	1.477	1.249	0.939	1.661	1.061	0.782	1.439	0.976	0.711	1.34	0.989	0.689	1.419	0.654	0.441	0.971
MILK (y vs, n)	0.881	0.803	0.966	0.823	0.736	0.92	0.766	0.687	0.854	0.747	0.678	0.823	0.769	0.697	0.848	0.766	0.699	0.839
SODA (n vs, y)	1.253	1.122	1.4	1.413	1.209	1.65	1.234	1.036	1.469	1.219	1.065	1.394	1.466	1.241	1.73	1.388	1.152	1.671
ALCOOL ABUSE (n vs, y)	1.018	0.921	1.126	0.971	0.859	1.098	0.937	0.827	1.062	0.842	0.736	0.962	0.803	0.693	0.93	0.833	0.718	0.966
POOR HEALTH SELF-RELATED (n vs, y)	2.011	1.382	2.927	2.441	1.533	3.886	2.529	1.695	3.773	1.566	1.122	2.186	1.839	1.342	2.52	1.888	1.462	2.438
HYPERTENSION (n vs, y)	1.167	0.958	1.423	1.113	0.955	1.297	1.014	0.902	1.14	1.819	1.408	2.35	1.183	1.027	1.362	1.15	1.044	1.267
DIABETES (n vs, y)	1.307	0.795	2.148	1.519	1.091	2.114	1.121	0.932	1.348	0.974	0.638	1.487	1.267	0.948	1.692	1.00	0.856	1.168
CRIME MORTALITY (1 vs, 3)	0.702	0.482	1.023	0.888	0.558	1.412	0.807	0.508	1.283	0.889	0.591	1.336	0.953	0.641	1.416	1.195	0.824	1.733
CRIME MORTALITY (2 vs, 3)	0.872	0.658	1.157	0.898	0.636	1.266	0.968	0.685	1.368	1.206	0.893	1.63	0.984	0.727	1.331	1.287	0.961	1.724
TRAFFIC ACCIDENT MORTALITY (1 vs, 3)	1.432	0.939	2.182	1.037	0.623	1.727	0.939	0.56	1.573	1.611	1.027	2.526	1.177	0.763	1.816	0.902	0.598	1.359
TRAFFIC ACCIDENT MORTALITY (2 vs, 3)	1.129	0.826	1.544	1.139	0.78	1.664	0.916	0.623	1.346	0.957	0.685	1.338	0.958	0.687	1.337	0.942	0.684	1.298
POPULATION (2 vs, 1)	1.346	0.582	3.112	0.63	0.212	1.869	1.388	0.427	4.51	0.327	0.12	0.895	0.917	0.339	2.479	0.372	0.134	1.031
POPULATION (3 vs, 1)	1.955	0.826	4.629	0.706	0.238	2.096	1.376	0.442	4.289	0.332	0.12	0.918	1.126	0.412	3.08	0.724	0.27	1.946
FEMALE PROPORTION (2 vs, 1)	0.776	0.419	1.437	1.736	0.787	3.828	1.034	0.445	2.404	1.875	0.921	3.815	0.955	0.473	1.928	1.679	0.829	3.401
FEMALE PROPORTION (2 vs, 1)	0.857	0.434	1.694	1.484	0.63	3.496	1.131	0.461	2.775	2.145	0.997	4.614	1.304	0.621	2.738	1.467	0.717	3.001
VEHICLE FLEET [1/100,000] (2 vs, 1)	1.009	0.332	3.069	2.576	0.662	10.032	0.427	0.105	1.737	1.401	0.384	5.117	0.795	0.238	2.651	1.948	0.565	6.719
VEHICLE FLEET [1/100,000] (3 vs, 1)	0.409	0.121	1.386	1.939	0.446	8.426	0.195	0.042	0.914	1.045	0.259	4.215	0.78	0.209	2.907	1.577	0.423	5.88
CAR FLEET [1/100,000] (2 vs, 1)	1.121	0.399	3.147	0.492	0.144	1.683	2.987	0.832	10.723	1.121	0.349	3.596	1.832	0.621	5.406	0.592	0.194	1.812
CAR FLEET [1/100,000] (3 vs, 1)	1.314	0.456	3.789	0.535	0.154	1.859	3.594	0.981	13.161	1.045	0.324	3.374	1.114	0.37	3.361	0.597	0.199	1.791
BUS FLEET [1/100,000] (2 vs, 1)	1.306	0.798	2.138	0.896	0.474	1.697	1.503	0.771	2.929	1.328	0.746	2.363	1.018	0.568	1.824	0.918	0.529	1.592
BUS FLEET [1/100,000] (3 vs, 1)	1.441	0.753	2.757	1.053	0.467	2.376	1.078	0.446	2.603	0.834	0.399	1.742	0.867	0.409	1.837	0.761	0.365	1.586
GINI (2 vs, 1)	1.158	0.817	1.642	0.949	0.622	1.447	1.073	0.689	1.673	0.967	0.667	1.402	1.306	0.899	1.898	1.092	0.764	1.562
GINI (3 vs, 1)	1.12	0.79	1.59	0.864	0.553	1.35	0.702	0.439	1.12	0.627	0.421	0.933	0.694	0.474	1.017	0.681	0.462	1.005
PRIVATE HEALTH INSURANCE [1/100,000] (2 vs, 1)	0.557	0.273	1.135	0.914	0.379	2.205	1.146	0.435	3.021	1.221	0.541	2.756	1.285	0.571	2.891	3.701	1.577	8.687
PRIVATE HEALTH INSURANCE [1/100,000] (3 vs, 1)	0.455	0.243	0.853	0.619	0.286	1.343	0.63	0.272	1.461	0.962	0.474	1.955	0.686	0.343	1.372	1.903	0.928	3.903
GDP PERCAPITA [BR$] (2 vs, 1)	1.632	0.757	3.52	0.869	0.345	2.192	1.015	0.367	2.808	1.109	0.487	2.523	1.151	0.507	2.61	0.64	0.279	1.468
GDP PERCAPITA [BR$] (3 vs, 1)	1.541	0.541	4.391	0.831	0.236	2.928	1.303	0.34	4.997	0.977	0.319	2.994	1.371	0.451	4.169	0.374	0.126	1.112
EMPLOYEES PHYSICAL ACTIVITY [inhabit rate] (2 vs, 1)	0.802	0.49	1.311	1.164	0.606	2.236	0.672	0.351	1.285	1.488	0.863	2.567	0.805	0.461	1.405	0.973	0.578	1.637
EMPLOYEES PHYSICAL ACTIVITY [inhabit rate] (3 vs, 1)	0.921	0.512	1.657	1.691	0.787	3.635	1.437	0.642	3.217	1.672	0.855	3.27	1.371	0.702	2.676	2.011	1.031	3.921
COMPANY PHYSICAL ACTIVITY [inhabit rate] (2 vs, 1)	1.418	0.795	2.53	1.262	0.641	2.483	1.385	0.688	2.789	0.807	0.438	1.488	1.607	0.902	2.863	0.879	0.49	1.577
COMPANY PHYSICAL ACTIVITY [inhabit rate] (3 vs, 1)	1.787	0.773	4.131	2.166	0.817	5.743	1.92	0.68	5.425	1.331	0.545	3.253	1.586	0.661	3.803	0.619	0.259	1.478

INSOLATION [hours] (2 vs, 1)	0.849	0.599	1.204	1.197	0.773	1.854	1.275	0.837	1.941	0.846	0.567	1.261	1.077	0.746	1.554	1.122	0.781	1.611
INSOLATION [hours] (3 vs, 1)	0.818	0.609	1.098	1.079	0.756	1.54	1.035	0.733	1.461	0.793	0.57	1.104	1.006	0.74	1.368	1.016	0.756	1.365
RAINFALL [mm3] (1 vs, 3)	1.021	0.751	1.387	1.032	0.717	1.487	1.331	0.922	1.923	0.925	0.661	1.294	1.071	0.772	1.485	1.141	0.849	1.534
RAINFALL [mm3] (1 vs, 2)	0.944	0.76	1.173	1.199	0.921	1.561	1.028	0.789	1.34	1.279	1.006	1.626	0.772	0.61	0.978	1.113	0.892	1.388
MAX TEMPERATURE [Celsius] (2 vs, 1)	1.327	0.82	2.148	1.488	0.822	2.693	1.284	0.717	2.299	1.266	0.739	2.168	1.108	0.649	1.892	1.033	0.646	1.652
MAX TEMPERATURE [Celsius] (2 vs, 1)	1.104	0.785	1.552	1.203	0.795	1.818	0.872	0.568	1.337	1.361	0.943	1.965	0.992	0.696	1.414	0.809	0.576	1.136
MIN TEMPERATURE [Celsius] (2 vs, 1)	1.101	0.726	1.67	1.533	0.924	2.543	1.72	1.038	2.851	0.907	0.57	1.444	1.088	0.695	1.703	1.992	1.313	3.022
MIN TEMPERATURE [Celsius] (2 vs, 1)	0.634	0.344	1.167	1.104	0.515	2.367	0.758	0.352	1.632	0.717	0.369	1.394	0.767	0.396	1.483	1.384	0.744	2.577
AVERAGE HUMIDITY [%] (1 vs, 2)	0.923	0.462	1.843	1.169	0.505	2.705	0.86	0.354	2.089	1.932	0.91	4.1	0.756	0.364	1.569	1.275	0.622	2.613
AVERAGE HUMIDITY [%] (1 vs, 3)	1.212	0.65	2.258	1.046	0.494	2.214	1.05	0.473	2.329	1.816	0.92	3.587	0.854	0.442	1.648	1.103	0.581	2.096
MIN HUMIDITY [%] (2 vs, 1)	0.95	0.695	1.298	0.978	0.664	1.439	0.597	0.413	0.862	0.925	0.653	1.311	0.828	0.59	1.164	0.688	0.503	0.942
MIN HUMIDITY [%] (3 vs, 1)	1.273	0.677	2.392	1.1	0.519	2.332	0.908	0.42	1.963	1.774	0.889	3.537	0.909	0.476	1.739	0.943	0.506	1.758
MAX HUMIDITY [%] (2 vs, 1)	1.27	0.815	1.979	1.275	0.72	2.26	1.832	1.033	3.248	0.857	0.518	1.418	1.167	0.706	1.93	1.098	0.678	1.778
MAX HUMIDITY [%] (3 vs, 1)	1.233	0.836	1.817	1.193	0.728	1.954	1.731	1.066	2.813	0.896	0.581	1.382	1.095	0.711	1.688	1.212	0.812	1.808
LIFE EXPECTANCY [years] (2 vs, 1)	0.789	0.508	1.226	1.053	0.603	1.837	1.097	0.649	1.857	0.908	0.563	1.464	0.845	0.518	1.379	0.752	0.478	1.181
LIFE EXPECTANCY [years] (3 vs, 1)	0.741	0.343	1.602	0.837	0.317	2.21	0.737	0.278	1.958	1.058	0.449	2.493	0.505	0.214	1.189	0.91	0.418	1.982
MALE LIFE EXPECTANCY [years] (2 vs, 1)	1.162	0.695	1.941	0.98	0.509	1.887	0.903	0.472	1.728	0.869	0.487	1.549	1.398	0.781	2.505	1.728	1.013	2.947
MALE LIFE EXPECTANCY [years] (3 vs, 1)	1.585	0.769	3.265	1.131	0.458	2.791	0.96	0.376	2.454	1.071	0.473	2.421	2.622	1.173	5.862	1.742	0.818	3.712
FEMALE LIFE EXPECTANCY [years] (2 vs, 1)	1.324	0.737	2.377	1.739	0.849	3.561	1.403	0.678	2.904	1.76	0.925	3.349	1.287	0.678	2.441	1.295	0.701	2.392
FEMALE LIFE EXPECTANCY [years] (3 vs, 1)	1.259	0.639	2.481	2.057	0.888	4.765	1.435	0.626	3.289	1.501	0.704	3.199	1.585	0.766	3.281	1.165	0.589	2.304
FHS PUBLIC HEALTH [n] (2 vs, 1)	1.00	0.635	1.573	1.061	0.598	1.882	0.892	0.509	1.562	0.816	0.503	1.322	0.851	0.52	1.393	0.905	0.58	1.412
FHS PUBLIC HEALTH [n] (3 vs, 1)	0.489	0.225	1.064	0.798	0.309	2.063	0.379	0.145	0.991	0.868	0.37	2.034	0.406	0.175	0.939	0.53	0.238	1.182
PRIMARY CARE COVERAGE [%] (2 vs, 1)	1.033	0.733	1.456	1.159	0.759	1.768	0.879	0.578	1.336	1.042	0.708	1.533	1.161	0.797	1.691	1.06	0.744	1.512
PRIMARY CARE COVERAGE [%] (2 vs, 1)	1.143	0.782	1.671	0.973	0.612	1.547	0.765	0.488	1.2	0.919	0.599	1.411	0.877	0.584	1.317	1.383	0.947	2.019
PUBLIC INVESTMENT* IN SPORTS AND LEISURE PERCAPITA [BR$] (2 vs, 1)	1.115	0.896	1.387	0.966	0.744	1.255	1.107	0.847	1.448	1.17	0.932	1.469	0.95	0.757	1.192	1.146	0.92	1.429
PUBLIC INVESTMENT* IN SPORTS AND LEISURE PERCAPITA [BR$] (3 vs, 1)	1.216	0.871	1.697	1.403	0.922	2.134	1.863	1.226	2.833	1.298	0.887	1.9	1.107	0.772	1.587	1.545	1.093	2.183
PUBLIC INVESTMENT* IN HEALTH PERCAPITA [BR$] (2 vs, 1)	1.052	0.609	1.816	0.586	0.299	1.145	0.293	0.145	0.594	0.665	0.364	1.216	0.669	0.373	1.199	0.899	0.503	1.608
PUBLIC INVESTMENT* IN HEALTH PERCAPITA [BR$] (3 vs, 1)	1.594	0.885	2.871	0.758	0.371	1.545	0.58	0.281	1.2	0.784	0.411	1.494	0.765	0.405	1.445	1.259	0.685	2.312
DRINKING WATER DISTRIBUITION [%] (2 vs, 1)	1.159	0.683	1.968	1.027	0.547	1.926	1.643	0.831	3.25	0.859	0.47	1.57	1.313	0.726	2.377	0.798	0.456	1.396
DRINKING WATER DISTRIBUITION [%] (3 vs, 1)	1.417	0.683	2.94	0.898	0.379	2.129	2.302	0.911	5.815	1.106	0.49	2.497	1.901	0.848	4.261	0.641	0.294	1.396
BASIC GROCERY PACKAGE [BR$] (1 vs, 3)	1.094	0.524	2.285	0.665	0.259	1.708	0.775	0.285	2.105	0.611	0.267	1.398	0.947	0.419	2.143	1.425	0.63	3.223
BASIC GROCERY PACKAGE [BR$] (2 vs, 3)	0.897	0.693	1.161	0.895	0.64	1.251	0.815	0.591	1.124	0.855	0.643	1.136	0.883	0.668	1.169	0.963	0.735	1.262
FAMILY INCOME < 1/2 MIN WAGE [%] (1 vs, 3)	1.138	0.586	2.209	0.521	0.227	1.199	0.513	0.22	1.196	0.972	0.45	2.097	0.96	0.466	1.977	0.656	0.323	1.333
FAMILY INCOME < 1/2 MIN WAGE [%] (2 vs, 3)	1.299	0.866	1.948	0.692	0.412	1.161	1.143	0.679	1.926	0.875	0.554	1.383	1.047	0.68	1.614	0.963	0.623	1.487
FAMILY INCOME 1/2 TO 1 MIN WAGE [%] (1 vs, 3)	0.72	0.482	1.077	1.634	0.977	2.732	1.392	0.84	2.306	1.106	0.704	1.738	1.426	0.918	2.217	0.917	0.603	1.395
FAMILY INCOME 1/2 TO 1 MIN WAGE [%] (3 vs, 3)	0.784	0.54	1.136	1.564	0.968	2.526	0.988	0.613	1.593	1.281	0.846	1.942	1.484	0.989	2.226	1.16	0.781	1.722
FAMILY INCOME 1 TO 2 MIN WAGE [%] (2 vs, 1)	1.948	0.909	4.176	0.942	0.358	2.481	1.287	0.448	3.702	0.962	0.386	2.395	0.765	0.307	1.909	0.847	0.337	2.127
FAMILY INCOME 1 TO 2 MIN WAGE [%] (3 vs, 1)	1.48	0.703	3.116	0.802	0.315	2.043	0.775	0.283	2.12	0.803	0.335	1.925	0.912	0.382	2.176	0.682	0.285	1.631
FAMILY INCOME > 2 MIN WAGE [%] (2 vs, 1)	1.447	0.862	2.431	1.354	0.736	2.489	0.749	0.397	1.413	1.545	0.867	2.753	0.663	0.377	1.163	1.138	0.675	1.917
FAMILY INCOME > 2 MIN WAGE [%] (3 vs, 1)	1.586	0.627	4.013	0.879	0.283	2.727	1.006	0.31	3.263	0.662	0.233	1.877	0.44	0.161	1.202	0.947	0.35	2.566
TV > 3 HOURS/DAY (n vs, y)	1.09	0.976	1.217	1.036	0.905	1.186	1.022	0.897	1.164	1.001	0.889	1.127	1.109	0.974	1.261	1.041	0.936	1.159
